# Translating the vaginal microbiome: gaps and challenges

**DOI:** 10.1186/s13073-016-0291-2

**Published:** 2016-04-01

**Authors:** Jacques Ravel, Rebecca M. Brotman

**Affiliations:** Institute for Genome Sciences, Department of Microbiology and Immunology, University of Maryland School of Medicine, Baltimore, MD USA; Department of Microbiology and Immunology, University of Maryland School of Medicine, Baltimore, MD USA; Department of Epidemiology and Public Health, University of Maryland School of Medicine, Baltimore, MD USA

## Abstract

Management, manipulation, and restoration of a robust vaginal microbiota has the potential to vastly improve women’s health and disease prevention. However, a systems level understanding of how the vaginal microbiota is associated with gynecologic and reproductive health is still needed to develop effective interventional strategies.

## A protective vaginal microbiota?

Decades of research have shown that the microbes inhabiting the human vagina (the vaginal microbiota) provide a first line of defense in the female reproductive tract. Women who are deficient in vaginal *Lactobacillus* spp. are at risk for serious and costly reproductive diseases and adverse obstetric outcomes. These diseases and adverse outcomes include the acquisition of sexually transmitted infections, such as human immunodeficiency virus (HIV) infections, and pre-term delivery, miscarriage, and pelvic inflammatory disease [1]. Projections indicate that up to 30 % of new HIV cases could be averted if the composition of the vaginal microbiota was dominated by robust, lactic acid-producing *Lactobacillus* spp. A paucity of *Lactobacillus* spp. in the vaginal microbiota can result in the clinical diagnosis of bacterial vaginosis (BV), an episodic, recurrent, symptomatic, and polymicrobial state that is the most common vaginal disorder in women of reproductive age. Despite the importance of the vaginal microbiota, surprisingly little is known about how it protects the female reproductive tract or any other roles a robust vaginal ecosystem performs. This knowledge gap represents a major challenge to the development of effective and practical clinical therapeutics that could protect and improve the health of large populations of women.

## Harnessing the protective features of the vaginal microbiota

Historically, the presence of *Lactobacillus* spp. has been thought to be the *sine qua non* of healthy vaginal microbial communities in reproductive-age women. These species, *L. crispatus, L. iners, L. gasseri,* and *L. jensenii*, seem to be specific to the human vagina, where they leverage a unique anaerobic nutritional environment to produce copious amounts of lactic acid as a fermentation product and a low protective pH (3.5–4). Lactic acid is a potent, broad-spectrum bactericide and virucide [2]. Therefore, it is not surprising that interventional efforts have been and are being made to restore vaginal health through the application of oral and vaginal probiotic formulations of *Lactobacillus* spp. A few studies have demonstrated a potential for probiotics to prevent numerous female reproductive tract infections. However, the results of these approaches have been modest to date and much work remains to be done before vaginal probiotics can be introduced into preventive and treatment guidelines. Some evidence suggests a woman’s endogenous microbiota could affect the success of probiotic applications [3] and future preventive and therapeutic approaches may need to include personalized probiotics.

A major challenge to developing effective preventive approaches to improve women’s health is that a large proportion of women who lack appreciable numbers of *Lactobacillus* spp. (10–42 % of women), while classified as at risk for urogenital infections, can remain asymptomatic for BV. Current guidelines from the US Centers for Disease Control do not support antibiotic treatment for these asymptomatic women [4]. In addition, a recent Cochrane systematic review concluded that existing evidence does not yet justify the use of the currently available formulations of *Lactobacillus* probiotics as an adjunctive or replacement therapy for BV [5]. Research is ongoing to identify accurate, inexpensive, and rapid diagnostic approaches that reflect the health of the vaginal microbiota, as well as strategies to rationally select the most useful probiotic species, strains, or combinations of these bacteria for maintaining healthy vaginal microbiota and preventing and treating BV. Researchers are also investigating probiotics mechanisms of action and whether it is necessary to have these species colonize the vagina or whether they can simply act temporarily to facilitate the restoration of a *Lactobacillus*-dominated and low pH microenvironment. The development of strategies to reseed the vagina with beneficial microorganisms is further complicated by the results of longitudinal studies that have shown that the vaginal microbiota in some women is highly dynamic, going through states during which *Lactobacillus* spp. are lacking [6]. These states vary in frequency and duration and are therefore associated with varying levels of risk for urogenital infections [6]. Interestingly, the vaginal microbiota becomes far more stable during pregnancy, although the level of stability depends in part on the composition of the microbiota before pregnancy and the lack of menses [7, 8]. Overall, the drivers of vaginal microbial community dynamics and instability are poorly understood. Our ability to exploit the protective features of the vaginal microbiota in the future will largely depend on the development of rapid microbiota characterization tools that could be predictive of a women’s vaginal microbiota longitudinal profiles and used to anticipate risk to urogenital infections and decide if restoring a protective vaginal microbial environment is necessary. A systems level approach is essential to assess all of the components of the vaginal microenvironment and their interactions with the genetics, metabolic activities, and immunity of the host and with the genetics and functions of the vaginal microbiota, as well as their interactions with extrinsic factors such as the behaviors, hygiene, and diet of the host.

## Shaping the vaginal microbiota

The human host is constantly struggling with its need to tolerate beneficial microbes while being able to distinguish and eliminate sometimes similar microbes that are pathogenic. We still have an incomplete understanding of this balancing act, which requires an evolved and complex recognition system at the mucosal surfaces that combines innate and adaptive immune responses. This delicate and sophisticated system is believed to be shaped early on in life through exposure to key microorganisms. Our immune system, which has a role in controlling the diversity and abundance of microorganisms in the body, is in some way also shaped by these microorganisms [9]. This circular framework has been ignored over the years and should be reconsidered so that we can try to maximize the success of novel translational approaches. We hypothesize that exposure to the ‘right’ microbiota at birth, or soon after birth, could be critical to developing and maintaining a lifelong healthy vaginal microbiota. In a recent study in which infants born by cesarean section were exposed to the mother’s vaginal microbiota at birth, this exposure led, at 30 days post-delivery, to partial reconstitution of the infant’s microbiota at most body sites to a composition similar to that of the microbiota of babies born vaginally; however, the vaginal microbiota of the newborn girls was not investigated [10]. It is important to stress that this work is highly preliminary as it involved only a small number of infants. Additional research is required to define which mothers would benefit from the practice and which vaginal microbiota have optimal beneficial effects before the practice of exposure to their mothers’ vaginal microbiota can be recommended for babies born by cesarean section.

The host–microbe relationship might also be regulated by host innate and adaptive immune factors, which could act against colonization by non-indigenous microbial strains and in some cases trigger symptoms such as those associated with BV. Under this paradigm, early exposure to a non-*Lactobacillus*-dominated vaginal microbiota could lead to host tolerance and a stable long-term association with this type of vaginal microbiota. This tolerance could explain the absence of BV symptoms in some women who lack vaginal *Lactobacillus* spp. Reshaping the vaginal microbiota in the reproductive years to one that is dominated by *Lactobacillus* spp. might then present many challenges, as colonization with other microbes, even those considered to be beneficial, might face an adversely trained immune system. Therefore, a one-solution-fits-all approach is very likely to fail for most women with a suboptimal at risk vaginal microbiota. A more personalized approach that matches the treatment to the vaginal microbiome and probably also to the genome of the host should be investigated.

Future interventions might need to take advantage of a window of opportunity at birth, or soon after delivery, to seed the vaginal microbiota of a newborn girl, particularly when her mother carries a vaginal microbiota identified as being associated with a high risk of urogenital infections or that is not very robust. Possibilities in this area involve modifying the mother’s vaginal microbiota during or even prior to pregnancy or exposing the newborn girl to beneficial natural [10] or synthetic vaginal microbiota or probiotic mixtures immediately after birth. The right intervention should help the newborn girl build tolerance to beneficial vaginal microbes. However, far more research is needed to first define whether each type of vaginal microbiota is beneficial or not and determine the role of a mother’s vaginal microbiota in the establishment of her daughter’s microbiota and its impact on the child’s future gynecologic, obstetric, and general health outcomes.

## Conclusions

In the near future, manipulation of the vaginal microbiota has the potential to change the way clinicians approach women’s health and preventive care. Although we understand the protective role of the vaginal microbiota as a whole, much detail remains to be clarified. Humans are highly unique among mammals in that women can carry *Lactobacillus* spp. as the dominant or minor member of the vaginal microbiota, which leads us to ask what roles other vaginal bacteria might have and how their manipulation might affect their ancillary functions. The interaction between microbiota at other body sites with the vaginal microbiota should also be considered and, just like the newly appreciated gut–brain axis, a gut–vagina axis might have an important role in women’s health. The next decade of systems biology and epidemiologic research on the vaginal microbiota is expected to lead to antibiotic-sparing strategies designed to manage, modulate, and restore a robust vaginal microenvironment and ultimately improve the health of women and their children.

## Abbreviations

BV: Bacterial vaginosis
HIV: Human immunodeficiency virus
